# Inference of Boolean Networks Using Sensitivity Regularization

**DOI:** 10.1155/2008/780541

**Published:** 2008-05-25

**Authors:** Wenbin Liu, Harri LÃ¤hdesmÃ¤ki, Edward R Dougherty, Ilya Shmulevich

**Affiliations:** 1 Institute for Systems Biology, Seattle, WA 98103, USA; 2 Institute of Signal Processing, Tampere University of Technology, Tampere 325035, Finland; 3 Department of Electrical and Computer Engineering, Texas A&M University, College Station, TX 33101, USA; 4 Computational Biology Division, Translational Genomics Research Institute, Phoenix, AZ 77843, USA; 5 College of Computer Science and Engineering, Wenzhou University, Wenzhou 85004, China

## Abstract

The inference of genetic regulatory networks from global measurements of gene expressions is an important problem in computational biology. Recent studies suggest that such dynamical molecular systems are poised at a critical phase transition between an ordered and a disordered phase, affording the ability to balance stability and adaptability while coordinating complex macroscopic behavior. We investigate whether incorporating this dynamical system-wide property as an assumption in the inference process is beneficial in terms of reducing the inference error of the designed network. Using Boolean networks, for which there are well-defined notions of ordered, critical, and chaotic dynamical regimes as well as well-studied inference procedures, we analyze the expected inference error relative to deviations in the networks' dynamical regimes from the assumption of criticality. We demonstrate that taking criticality into account via a penalty term in the inference procedure improves the accuracy of prediction both in terms of state transitions and network wiring, particularly for small sample sizes.

## 1. Introduction

The execution of various developmental and physiological processes in cells is carried out by complex biomolecular systems. Such systems are dynamic in that they are able to change states in response to environmental cues and exhibit multiple steady states, which define different cellular functional states or cell types.

The massively parallel dynamics of complex molecular networks furnish the cell with the ability to process information from its environment and mount appropriate responses. To be able to stably execute cellular functions in a variable environment while being responsive to specific changes in the environment, such as the activation of immune cells upon exposure to pathogens or their components, the cell needs to strike a balance between robustness and adaptability.

Theoretical considerations and computational studies suggest that many types of complex dynamical systems can indeed strike such an optimal balance, under a variety of criteria, when they are operating close to a critical phase transition between an ordered and a disordered dynamical regime [1–3]. There is also accumulating evidence that living systems, as manifestations of their underlying networks of molecular interactions, are poised at the critical boundary between an organized and a disorganized state, indicating that cellular information processing is optimal in the critical regime, affording the cell with the ability to exhibit complex coordinated macroscopic behavior [4–8]. Studies of human brain oscillations [9], computer network traffic and the Internet [10,11], financial markets [12], forest fires [13], neuronal networks supporting our senses [14], and biological macroevolution have also revealed critical dynamics [15].

A key goal in systems biology research is to characterize the molecular mechanisms governing specific cellular behaviors and processes. This typically entails selecting a model class for representing the system structure and state dynamics, followed by the application of computational or statistical inference procedures for revealing the model structure from measurement data [16]. Multiple types of data can be potentially used for elucidating the structure of molecular networks, such as transcriptional regulatory networks, including genome wide transcriptional profiling with DNA microarrays or other high-throughput technologies, chromatin immunoprecipitation-on-chip (ChIP-on-chip) for identifying DNA sequences occupied by specific DNA binding proteins, computational predictions of transcription factor binding sites based on promoter sequence analysis, and other sources of evidence for molecular interactions [17,18]. The inference of genetic networks is particularly challenging in the face of small sample sizes, particularly because the number of variables in the system (e.g., genes) typically greatly outnumbers the number of observations. Thus, estimates of the errors of a given model, which themselves are determined from the measurement data, can be highly variable and untrustworthy.

Any prior knowledge about the network structure, architecture, or dynamical rules is likely to improve the accuracy of the inference, especially in a small sample size scenario. If biological networks are indeed critical, a key question is whether this knowledge can be used to improve the inference of network structure and dynamics from measurements. We investigated this question using the class of Boolean networks as models of genetic regulatory networks.

Boolean networks and the more general class of probabilistic Boolean networks are popular approaches for modeling genetic networks, as these model classes capture multivariate nonlinear relationships between the elements of the system and are capable of exhibiting complex dynamics [5,16,19–21]. Boolean network models have been constructed for a number of biomolecular systems, including the yeast cell cycle [22,23], mammalian cell cycle [24], Drosophila segment polarity network [25], regulatory networks of *E. coli* metabolism [26], and Arabidopsis flower morphogenesis [27–29].

At the same time, these model classes have been studied extensively regarding the relationships between their structure and dynamics. Particularly in the case of Boolean networks, dynamical phase transitions from the ordered to the disordered regime and the critical phase transition boundary have been characterized analytically for random ensembles of networks [30–34]. This makes these models attractive for investigating the relationships between structure and dynamics [35,36].

In particular, the so-called average sensitivity was shown to be an order parameter for Boolean networks [31]. The average sensitivity, which can be computed directly from the Boolean functions specifying the update rules (i.e., state transitions) of the network, measures the average response of the system to a minimal transient perturbation and is equivalent to the Lyapunov exponent [33]. There have been a number of approaches for inferring Boolean and probabilistic Boolean networks from gene expression measurement data [20,21,37–44].

We address the relationship between the dynamical regime of a network, as measured by the average sensitivity, and the inference of the network from data. We study whether the assumption of criticality, embedded in the inference objective function as a penalty term, improves the inference of Boolean network models. We find that for small sample sizes the assumption is beneficial, while for large sample sizes, the performance gain decreases gradually with increasing sample size. This is the kind of behavior that one hopes for when using penalty terms.

This paper is organized as follows. In Section 2, we give a brief definition of Boolean Networks and the concept of sensitivity. Then in Section 3, three measures used in this paper to evaluate the performance of the predicted networks are introduced, and a theoretical analysis of the relationship between the expected error and the sensitivity deviation is presented. Based on this analysis, an objective function is proposed to be used for the inference process in Section 4, while the simulation results are presented in Section 5.

## 2. Background and Definitions

### 2.1. Boolean Networks

A Boolean network  is defined by a set of nodes ,  and a set of Boolean functions , . Each node  represents the expression state of the gene , where  means that the gene is OFF, and  means it is ON. Each Boolean function  with  specific input nodes is assigned to node  and is used to update its value. Under the synchronous updating scheme, all genes are updated simultaneously according to their corresponding update functions. The network's state at time  is represented by a vector  and, in the absence of noise, the system transitions from state to state in a deterministic manner.

### 2.2. Sensitivity

The activity of gene  in function  is defined as(1)

where  is the partial derivative of  with respect to ,  is addition modulo 2, and , [31]. Note that the activity is equivalent to the expectation of the partial derivative with respect to the uniform distribution. Since the partial derivative is itself a Boolean function, its expectation is equal to the probability that a change in the th input causes a change in the output of the function, and hence the activity is a number between zero and one. The average sensitivity of a function  equals the sum of the activities of its input variables:(2)

In the context of random Boolean networks (RBNs), which are frequently used to study dynamics of regulatory network models, another important parameter is the bias  of a function , which is defined to be the probability that the function takes on the value 1. A random Boolean function with bias  can be generated by flipping a -biased coin  times and filling in the truth table. In other words, the truth table is a realization of  independent Bernoulli () random variables. For a function  with bias  the expectation of its average sensitivity is(3)

The sensitivity of a Boolean network is then defined as(4)

Sensitivity is in fact a global dynamical parameter that captures how a one-bit perturbation spreads throughout the network and its expectation under the random Boolean network model is equivalent to the well-known phase transition curve [31].

## 3. Error Analysis

### 3.1. Performance Measures

There are several ways to measure the performance of an inference method by comparing the state transitions, wiring, or sensitivities with the original network. In this paper, we will use three measures that are described below.

### 3.1.1. The State Transition Error

This quantity generally shows the fraction of outputs that are incorrectly predicted, and it can be defined as(5)

where  denotes the normalized error of the predicted function . Additionally,  and  are extended such that they are functions of all the variables (instead of  variables) by adding fictitious (i.e., dummy) variables.

### 3.1.2. The Receiver Operating Characteristic (ROC)

This measurement has been widely used in classification problems. An ROC space is defined by the false positive ratio (FPR) and the true positive ratio (TPR) plotted on the - and -axes, respectively, which depicts the relative tradeoffs between true positives and false positives. The FPR and TPR are defined as(6)

where TP and FP represent true positive and false positive instances, respectively, while  and  represent the total positive and negative instances, respectively. We will use the ROC distributions to evaluate the accuracy of "wiring" (i.e., the specific input nodes assigned to each node) for the inferred network.

### 3.1.3. The Sensitivity Error

The sensitivity error measures the deviation in the sensitivity of a predicted network and is defined as(7)

where  is the sensitivity of the predicted network.

### 3.2. Analysis of Expected Error

All Boolean networks with a fixed number of genes  can be grouped into different families according to the network's sensitivity. Assuming that  and  are the original network and another random network,  and  are their sensitivities, respectively, and  and  are the biases with which functions  and  are generated. Let . The expectation of the state transition error between them can be written as(8)

Using the relationship between sensitivity and bias in Section 2.2, we have(9)

Then,(10)

If we further assume that both networks' connectivity is constant,  (), then(11)

This means that the expectation of the state transition error  generally depends on the original network's connectivity , its sensitivity , the sensitivity deviation , and the mean quadratic terms of the bias deviation .

(1) If , then  will be 0. In this case, each function  keeps the same bias with that of the original network, and then(12)

(2) If  and  is 0, the predicted network still stays in the same sensitivity class, and(13)

(3) If  is relatively small compared with , we can treat it as a constant . Then,(14)

In this case,  will have a linear relationship with . This indicates that  will surely introduce additional error . Our simulations indicate that the inference method we use (best-fit, see below) yields a network with  in most cases.

## 4. Inference Method

To infer a Boolean network, for each target node we need to apply some optimization criterion to each set of input variables and Boolean function on those input variables and then choose the variable set and corresponding Boolean function that minimizes the objective function. The first step in our proposed procedure is to find variable sets and Boolean functions that provide good target prediction. Based upon time-series observations, given a target node  and an input-node vector  the best predictor, , minimizes the error, , among all possible predictors . Finding the best predictor for a given node means finding the minimal error among all Boolean functions over all input-variable combinations. We consider three variable combinations. Since we will optimize via an objective function containing a sensitivity penalty term, we will select a collection of input-variable sets and select the minimal-error Boolean function over each input-variable set. This is accomplished by using the plugin (resubstitution) estimate of the error , which is given by the number of times  in the data divided by the number of times, the pair  is observed in the data. This procedure is equivalent to the best-fit extension method [45]. We make use of an efficient algorithm for solving the best-fit extension problem and finding all functions having error smaller than a given threshold [37]. We then select the four best variable sets and corresponding Boolean functions as candidates. The limitation of four candidates is based on computational considerations; in principle, there is no such limitation.

Because we have a small data sample, if we were to use the resubstitution error estimates employed for variable selection as error estimates for the best Boolean functions, we would expect optimistic estimates. Hence, for each selected variable set, we estimate the error of the corresponding Boolean function via the .632 bootstrap [46,47]. A bootstrap sample consists of  equally likely draws with replacement from the original sample consisting of  data pairs . For the zero-bootstrap estimator, , the function is designed on the bootstrap sample and tested on the points left out, this is done repeatedly, and the bootstrap estimate is the average error made on the left-out points.  tends to be a high-biased estimator of the true error, since the number of points available for design is on average only 0.632 . The .632 bootstrap estimator attempts to correct this bias via a weighted average,(15)

where  is the original resubstitution estimate.

We summarize the procedure as follows.

(1) For each three-variable set , do the following.

(i) Compute the resubstitution errors of all Boolean functions using the full sample data set.

(ii) Choose the Boolean function, , possessing the lowest resubstitution error as the corresponding function for .

(iii) Bootstrap the sample and compute the zero-bootstrap error estimate for .

(iv) Compute the .632 bootstrap error estimate for  using the computed resubstitution and zero-bootstrap estimates.

(2) Select the four input-variable sets whose corresponding functions possess the lowest .632 bootstrap estimates.

This procedure is the same as the one used in [48] to evaluate the impact of different error estimators on feature-set ranking. It was demonstrated there that the bootstrap tends to outperform cross-validation methods in choosing good feature sets. While it was also observed that bolstering tends to be slightly better than bootstrap, bolstering cannot be applied in discrete settings, so it is not a viable option in our context.

Motivated by the analysis in Section 3, we refine the inference process by incorporating the sensitivity. We construct an objective function(16)

where  represents the bootstrap-estimated error of the previously selected Boolean function and  is the sensitivity error. The first item represents the prediction error, while the second represents the "structural error" associated with general network dynamics. Our hypothesis is that a better inference should have a small error in both state transition and sensitivity, and consequently, the value of its objective function  should be minimal. Of the four input-variable sets selected via prediction error for a target node, we use the one with minimal objective function  for network construction.

## 5. Simulation Results

All simulations are performed for random Boolean networks with  and . For a given BN, we randomly generate  pairs of input and output states. We also consider the effect of noise, with 5% noise added to the output states of each gene by flipping its value with probability 0.05.

From the perspective of network inference, performance is best characterized via a distance function between the ground-truth network and the inferred network, more specifically, by the expected distance between the ground-truth and inferred network as estimated by applying the inference to a random sample of ground-truth networks [49]. In our case, we have chosen the normalized state-transition error as the distance between the networks.

**Figure 1:**
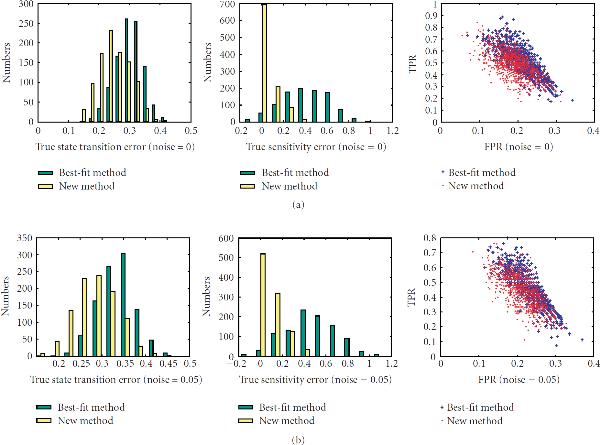
Histograms of the true error in state transition and in sensitivity and the ROC distribution for the 1000 random BNs under sample size 10.

**Figure 2:**
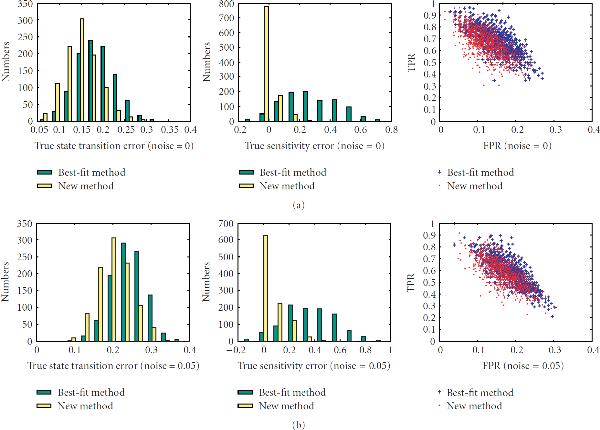
**Histograms of the true error in state transition and in sensitivity and the ROC distribution for the 1000 random BNs under sample size 15**.

**Figure 3:**
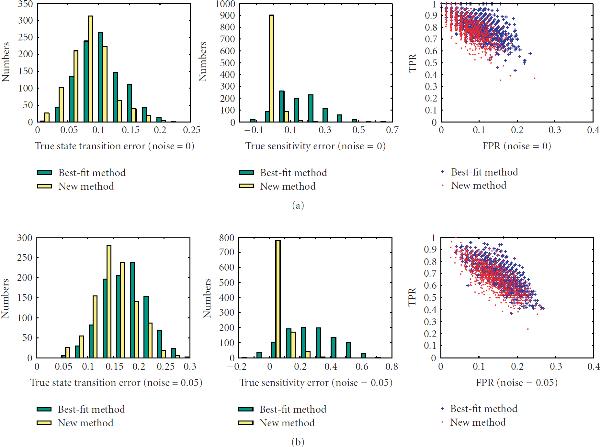
**Histograms of the true error in state transition and in sensitivity and the ROC distribution for the 1000 random BNs under sample size 20**.

**Figure 4:**
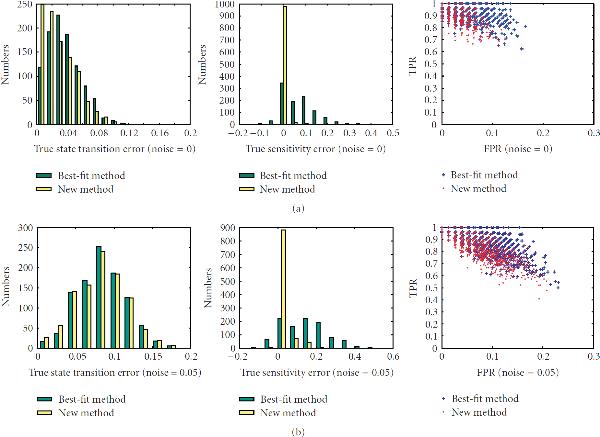
**Histograms of the true error in state transition and in sensitivity and the ROC distribution for the 1000 random BNs under sample size 30**.

**Figure 5:**
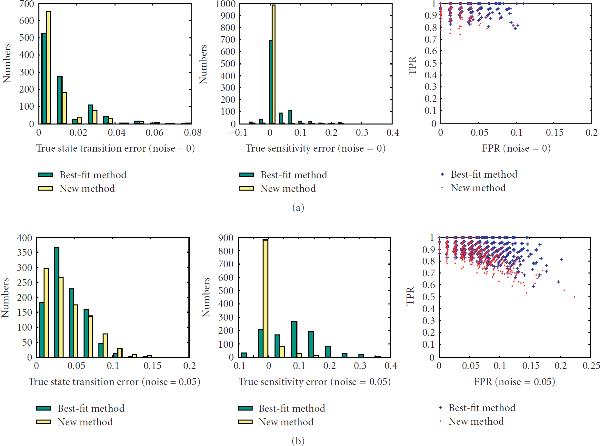
**Histograms of the true error in state transition and in sensitivity and the ROC distribution for the 1000 random BNs under sample size 40**.

**Figure 6:**
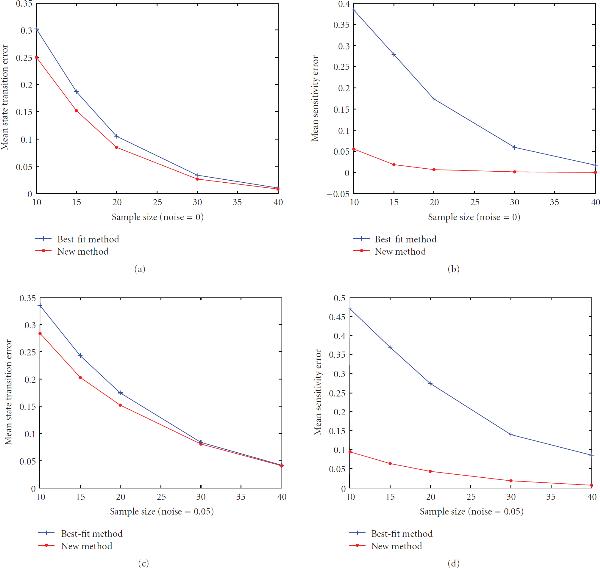
**Mean state transition and sensitivity error for sample sizes ranging from 10 to 40, computed with zero noise and 5% noise**.

First, we investigate the performance of the new method on networks with different sensitivities, , on sample sizes ranging from 10 to 40. There are total of 200 networks for each value of the sensitivity. The left columns of Figures 1–5 are the histograms of the distribution of the true state-transition error (Section 3.1.1) for both the traditional best-fit method (combined with .632 bootstrap) and the new proposed method. They show that the proposed method reduces this error dramatically in small sample situations. As sample size increases, the performance of both methods becomes closer. The middle columns of Figures 1–5 are the histograms of the distribution of the sensitivity error (Section 3.1.3). As can be seen, the best-fit method usually ends up with a network with larger sensitivity in small sample cases, while the proposed method can find a network operating in the same or nearby dynamic regime. The right columns of Figures 1–5 are the ROC distributions of both methods (Section 3.1.2). The proposed method has approximately the same *TPR* as the best-fit method but with a lower *FPR*. This means that the recovered connections will have higher reliability. Figure 6 shows the mean error in state transition and sensitivity under different samples sizes, for zero noise and 5% noise.

**Figure 7:**
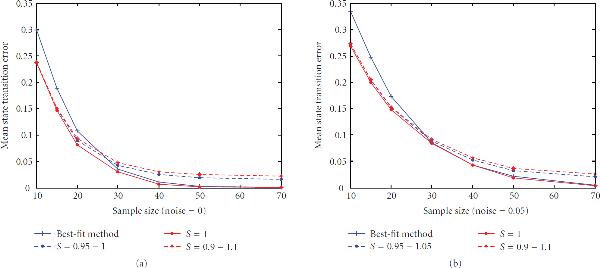
**Mean state transition error of different sensitivity deviation for sample sizes ranging from 10 to 70, computed with zero noise and 5% noise**.

In practice, we do not know the network sensitivity, so that the assumed value in the inference procedure may not agree with the actual value. Hence, the inference procedure must be robust to this difference. Under the assumption of criticality for living systems, it is natural to set  in the inference procedure. Moreover, assuming that a living system remains near the border between order and disorder, the true sensitivity of gene regulatory networks will remain close to 1 under the Boolean formalism. Thus, to investigate robustness, we generated 1000 networks with sensitivities , and then inferred them using  with the proposed method. The mean state transition errors of both methods are shown in Figure 7.

When the actual sensitivity is 1, the method helps for small samples and the performances become close for large samples, analogous to Figure 6. When the true network deviates from the modelling assumption, , the proposed method helps for small samples and results in some loss of performance for large samples. This kind of behavior is what one would expect with an objective function that augments the error. In effect, the sensitivity is a penalty term in the objective function that is there to impose constraint on the optimization. In our case, when the true sensitivity is not equal to 1, the sensitivity constraint  yields smaller sensitivity error than the best-fit method in small sample situations, while the sensitivity error of the best-fit method is smaller for large samples. In sum, the constraint is beneficial for small samples.

**Figure 8:**
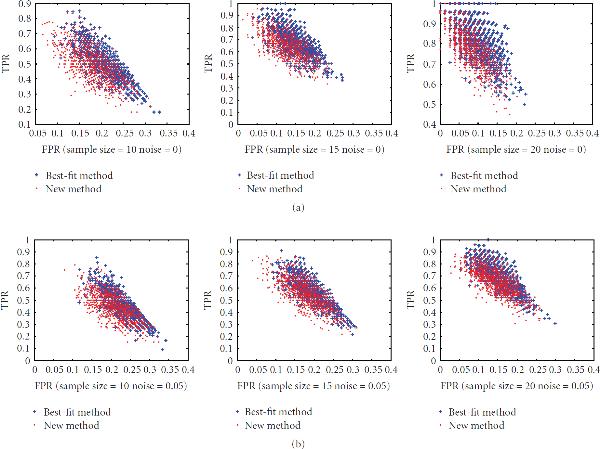
**The distribution of ROC with sensitivity deviation from 0.9 to 1.1 for sample sizes 10, 15, and 20, computed with zero noise and 5% noise**.

Finally, the performance of the new method with regard to wiring for small sensitivity deviation is presented in Figure 8. It shows that the new method can achieve the same *TPR* with a lower *FPR* under a small sensitivity deviation in small sample situations.

## 6. Conclusions

Sensitivity is a global structural parameter of a network which captures the network's operating dynamic behavior: ordered, critical, or chaotic. Recent evidence suggests that living systems operate at the critical phase transition between ordered and chaotic regimes. In this paper, we have proposed a method to use this dynamic information to improve the inference of Boolean networks from observations of input-output relationships. First, we have analyzed the relationship between the expectation of the error and the deviation of sensitivity, showing that these quantities are strongly correlated with each other. Based on this observation, an objective function is proposed to refine the inference approach based on the best-fit method. The simulation results demonstrate that the proposed method can improve the predicted results both in terms of state transitions, sensitivity, and network wiring. The improvement is particularly evident in small sample size settings. As the sample size increases, the performance of both methods becomes similar. In practice, where one does not know the sensitivity of the true network, we have assumed it to be 1, the critical value, and investigated inference performance relative to its robustness to the true sensitivity deviating from 1. For small samples, the kind we are interested in when using such a penalty approach, the proposed method continues to outperform the best-fit method.

For practical applications, one can apply an optimization strategy, such as genetic algorithms, to attain suboptimal solutions instead of the brute force searching strategy used in this paper. As the final chosen function for each gene generally lies within the top three candidates in our simulations, one can just select from a few top candidate functions for each gene instead of using all of the possible  candidates. Finally, it should be noted that the ideas presented here could also be incorporated into other inference methods, such as the ones in [40,41].
